# Case Report: Application of the AngioJet thrombectomy system in acute lower extremity deep vein thrombosis complicated by transplanted renal vein thrombosis (report of two cases)

**DOI:** 10.3389/fcvm.2025.1513776

**Published:** 2025-09-15

**Authors:** Lifan Shao, Guangxin Cao, Suiyuan Shang, Bo Sun, Wuguang Ji, Jiefeng Zhang, Tao Liu

**Affiliations:** ^1^ School of Clinical, Shandong Second Medical University, Weifang, Shandong, China; ^2^ Weifang People's Hospital, Weifang, Shandong, Department of Vascular Surgery, Weifang, Shandong, China

**Keywords:** transplanted renal vein thrombosis, deep vein thrombosis (DVT), kidney transplantation, AngioJet thrombectomy system, endovascular therapy

## Abstract

Lower extremity deep venous thrombosis (DVT) combined with transplanted renal vein thrombosis represents a rare and complex form of venous thromboembolism that leads to obstruction of the transplanted renal vein. This condition results in parenchymal edema of the kidney, ultimately impairing the function of the transplanted organ. It constitutes a catastrophic complication following renal transplantation, potentially resulting in loss of function of the transplanted kidney and failure of the surgical procedure. The primary objective of treatment is to promptly remove thrombi from the transplanted renal vein, thereby restoring normal venous return and renal function as swiftly as possible to enhance patient prognosis. Currently, surgical thrombectomy and thrombolytic therapy are considered the mainstay treatment modalities. Surgical thrombectomy is generally recommended as a first-line approach due to its efficacy in achieving rapid thrombus removal. To date, there exists limited literature regarding the utilization of the AngioJet thrombectomy system for managing DVT in conjunction with transplanted renal vein thrombosis. In this report, we present two cases involving middle-aged male patients diagnosed with acute lower extremity DVT complicated by transplanted renal vein thrombosis. Both patients had undergone allogeneic kidney transplantation 15 and 4 years prior, respectively. In these instances, we employed the AngioJet thrombectomy system for emergency thrombus aspiration treatment. The thrombi within both patients' lower extremity deep veins and their respective transplanted renal veins were completely removed. Subsequently, urine output gradually increased for both patients; moreover, their renal function progressively improved to an acceptable range. Notably, neither patient developed postoperative complications nor exhibited any recurrence of thrombi during follow-up evaluations.

## Case 1

**Figure 1:**
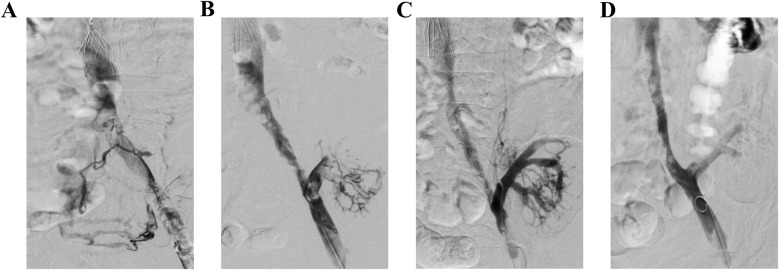
Angiography in operation. **(A)** Angiography revealed a fresh thrombosis in the left iliac vein. **(B)** The main and branch thrombi of the transplanted kidney vein can be seen after AngioJet thrombectomy of the left iliac vein. **(C** and **D)** The main and branches of the transplanted renal vein were well developed after AngioJet thrombectomy and the iliac vein was clear.

**Figure 2:**
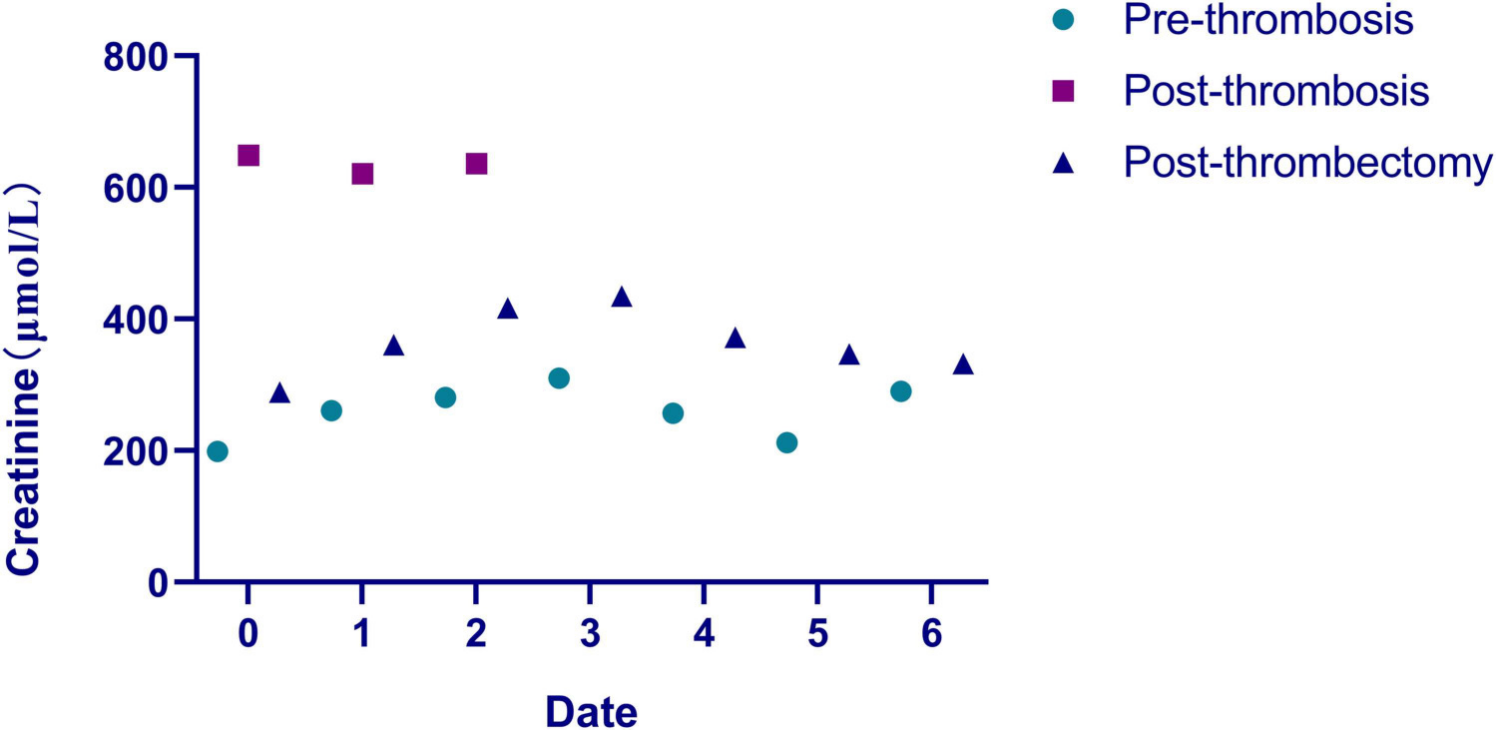
The serum creatinine value trend before and after surgery. Round dots: serum creatinine levels before transplant renal vein thrombosis formation; square dots: creatinine values post-thrombosis but pre-AngioJet thrombectomy; triangle dots: post-AngioJet thrombectomy creatinine measurements.

A 41-year-old man with a history of allogeneic kidney transplantation performed 15 years prior was admitted to our hospital due to an acute onset of left lower abdominal pain and discomfort, accompanied by significant swelling of the left lower limb lasting for 2 days. During this period, his urine output decreased sharply from approximately 1,500–2,000 to 200 mL/day. Color Doppler ultrasound indicated thrombosis from the left common femoral vein to the left common iliac vein. In addition, it indicated thrombosis formation in the left transplanted kidney vein. The results of the laboratory examination are as follows: D-dimer: 60.95 μg/mL (reference value 0–0.55 μg/mL); creatinine rose sharply from 240 μmol/L before onset to 649 μmol/L (reference value 51–97 μmol/L). Suspecting acute kidney injury of the transplanted kidney, the patient underwent surgical treatment 4 h after admission. First, an inferior vena cava (IVC) filter was inserted emergently to prevent the thrombus from moving and subsequent fatal pulmonary embolism. Then, an AngioJet thrombus aspiration catheter was used to perform a thrombectomy in the left iliac vein, left femoral vein, and transplanted kidney vein. A 5 F curved catheter with negative pressure was used to manually aspirate the thrombus from the branch of the transplanted renal vein. Angiography confirmed patency restoration of the left external iliac and common femoral veins, with no residual thrombus observed in the main trunk or branches of the transplanted renal vein (Figure 1). However, approximately 50% stenosis remained in the left common iliac vein post-thrombus removal. Then, after ATLAS balloon (16 mm × 60 mm) dilatation of the left common iliac vein, re-angiography showed that the blood supply of the left common iliac vein was significantly improved. The blood flow velocity and residual stenosis (30%) were acceptable without stent graft implantation. The patient underwent continuous renal replacement therapy (CRRT) for 3 days post-procedure. Medical management included immunosuppressants (tacrolimus, mycophenolate mofetil, and prednisone) and anticoagulation with low-molecular-weight heparin (LMWH) (enoxaparin 80 U/kg every 12 h). Clinical improvement was subsequently noted, with urine output progressively increasing to approximately 1,500 mL/day and renal function showing recovery, as evidenced by a decrease in serum creatinine to around 332 μmol/L. After discharge, anticoagulation therapy (rivaroxaban 20 mg q.d.) was prescribed for 3 months. At the 3-month follow-up, the patient's renal function had remained stable, with serum creatinine maintained around 300 μmol/L and urine output consistently between 1,500 and 2,000 mL/day (Figure 2). Postoperative surveillance with Doppler ultrasound at 1- and 3-month intervals confirmed patency without thrombosis or stenosis in the iliac venous system.

## Case 2

**Figure 3:**
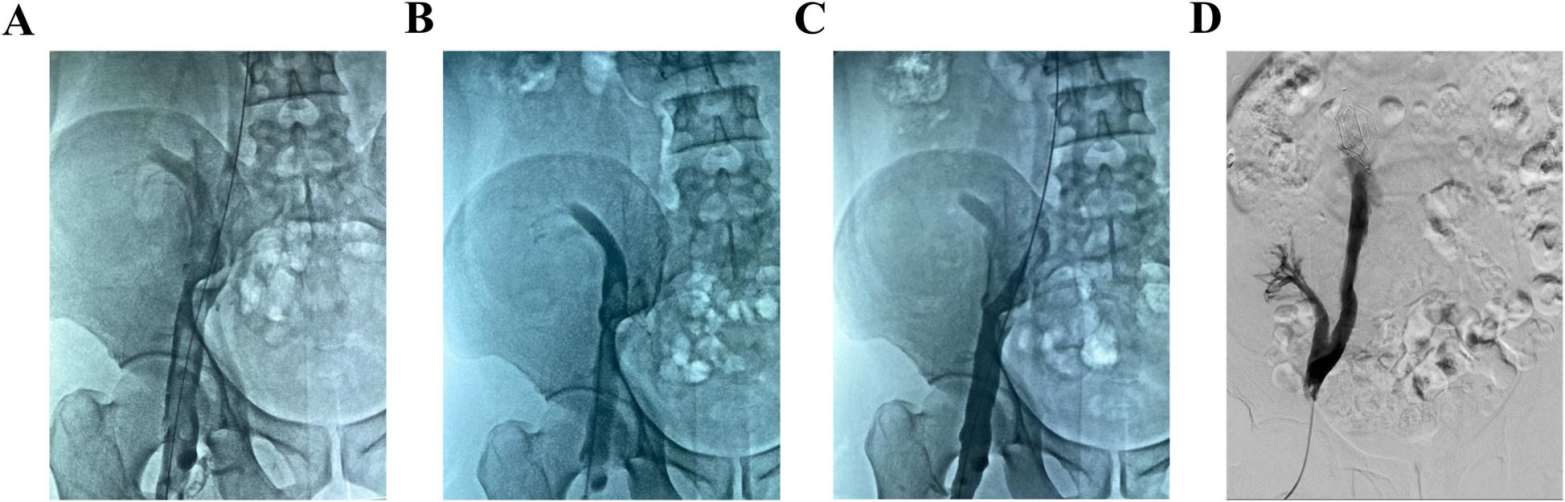
Angiography in operation. **(A)** Angiography revealed filling defects in the right iliac and femoral veins, suggesting the formation of a new thrombus. **(B)** After AngioJet thrombectomy, the main thrombus of the external iliac vein and femoral vein transplantation of the kidney vein was cleared, but the common iliac vein was still not clear. **(C)** Angiography showed that there was still a thrombus in the branch of the transplanted kidney vein. **(D)** After balloon dilatation, the right femoral vein, external iliac vein, common iliac vein, and transplanted kidney vein were shown to be well developed, with unobtrusive blood flow and no obvious filling defect.

**Figure 4:**
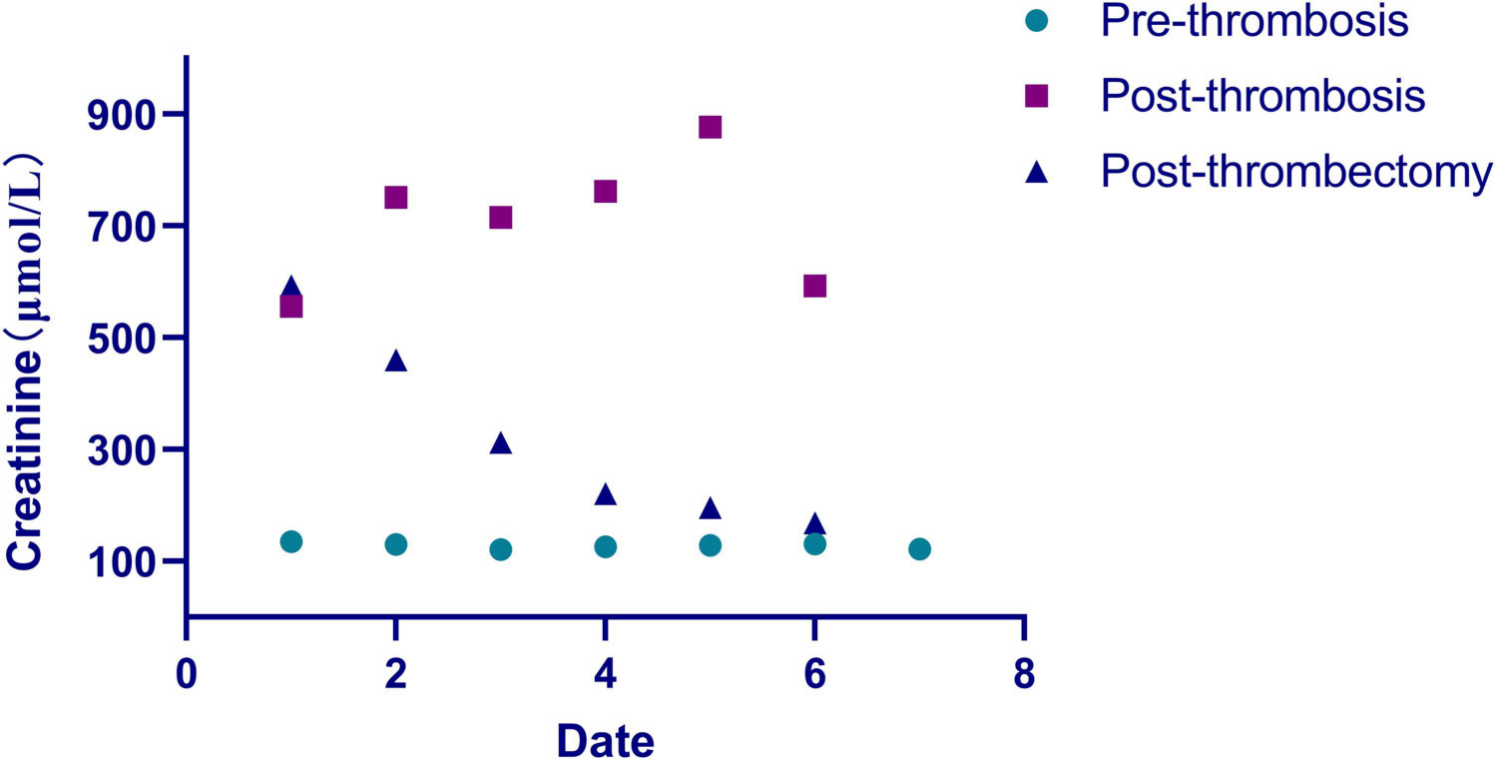
The serum creatinine value trend before and after surgery. Round dots: serum creatinine levels before transplant renal vein thrombosis formation; square dots: creatinine values post-thrombosis but pre-AngioJet thrombectomy; triangle dots: post-AngioJet thrombectomy creatinine measurements.

A 47-year-old male patient underwent allogeneic kidney transplantation at our hospital due to end-stage chronic renal failure 5 years prior. The patient presented with sudden swelling and pain in the right lower limb of unclear etiology. Color Doppler ultrasonography demonstrated thrombosis formation of the right iliac vein, femoral vein, and transplanted renal vein. Meanwhile, the patient developed anuria and hypercreatininemia (751 μmol/L). IVC filter placement and AngioJet thrombectomy were performed at 5.5 h post-admission. The venography showed thrombosis in the iliofemoral vein and the transplanted kidney vein (Figure 3A). After using an 8 F AngioJet thrombus aspiration catheter to aspirate the thrombus, the re-angiography indicated good recanalization of the right femoral vein, external iliac vein, and transplanted kidney vein (Figure 3B). However, the angiographic evaluation indicated severe stenosis of the right common iliac vein (>80%) (Figure 3C). Subsequently, a MUSTANG balloon (10 mm × 60 mm) and an ATLAS balloon (14 mm × 60 mm) were applied to dilate the common iliac vein. The stenosis of the common iliac vein was significantly improved after the dilation (residual stenosis rate<10%) (Figure 3D). Postoperatively, CRRT was maintained for 3 days, alongside ongoing anti-rejection therapy with tacrolimus, mycophenolate mofetil, and prednisolone. During hospitalization, low-molecular-weight heparin (80 U/kg every 12 h) was administered for routine anticoagulation. The patient's renal function gradually recovered, with serum creatinine dropping to 169 μmol/L (Figure 4) and urine volume recovering to 2,000 mL/day. After discharge, the anticoagulant therapy was prescribed with rivaroxaban (20 mg/day) for at least 3 months. The function of the transplanted kidney was good after 15 months of follow-up, with a urine volume of approximately 2,000 mL/day and creatinine stable at approximately 160 μmol/L. Postoperative surveillance with Doppler ultrasound at 1-, 3-, and 6-month intervals confirmed patency without thrombosis or stenosis in the iliac venous system.

## Discussion

Acute deep venous thrombosis (DVT) of the lower limbs is among the most common vascular emergencies. The pathogenesis is primarily explained by Virchow's triad, which includes vascular wall injury, stasis of blood flow, and hypercoagulability (1, 2). In clinical practice, most DVT cases are caused by abnormal anatomical structure and internal environmental factors, with clinical decision-making based on the cause. There is a consensus on the treatment of patients with lower extremity DVT and numerous clinical guidelines have been developed. However, several DVT types are relatively rare in clinical practice and require interdisciplinary consultation. For these cases, there are few guidelines or expert recommendations that can be followed and case reports exploring viable modalities are necessary.

Most cases of organ failure in transplanted kidneys result from rejection and inflammation; however, vascular factors are a rare cause and can only be diagnosed after the exclusion of the common causes. Among these, the condition of the iliac and femoral arteries and veins can affect the prognosis of patients (3). A common cause is anastomotic stenosis of the transplanted kidney. However, in patients with a kidney transplant and renal insufficiency caused by renal vein thrombosis, the clinical symptoms lack specificity. Although the two cases in this article showed decreased urine volume, which indicated decreased kidney function, it could not be associated with a vascular cause until the presence of sudden lower limb edema combined with reduced urine volume. Finally, a definitive diagnosis was established through a color Doppler ultrasound examination, which revealed the presence of thrombosis in the transplanted venous vein.

DVT of the lower extremities complicated by renal vein thrombosis represents a complex pathogenic process influenced by multiple factors. The reported incidence rate in previous studies ranges from approximately 0.1% to 4.2% (4), and usually leads to irreversible kidney damage and kidney transplant failure (5). Mechanical factors are considered the most common cause of renal vein thrombosis after transplantation (6), such as anastomotic stenosis of the renal vein, iliac vascular graft compression, May–Thuner syndrome (7), and the influence of inferior vena cava filters (8). Other factors include long-term oral administration of hormones and other anti-rejection drugs, inherited thrombophilia (9), malignancy (10), and pelvic adhesion. Therefore, the formation of thrombus in renal graft patients is often dominated by one factor and the synergistic action of other factors. In these two reported cases, compression and stenosis of the common iliac vein were found during the operation, which acted as the initial cause of further thrombosis. The transplanted renal vein was patent, and no stenosis or distortion was found in the angiography. Therefore, we propose that the vascular surgery department should evaluate the patency degree of the deep veins in the lower limbs in advance before kidney transplantation for the position of the anastomotic blood vessel of the transplanted kidney. This approach would avoid deep vein thrombosis of the lower limbs secondary to iliac vein stenosis, which would then lead to the spread of the transplanted kidney vein thrombosis. According to reports in the literature, doppler ultrasound (DUS) or angiography are effective methods (9, 11). Furthermore, the prolonged use of immunosuppressive regimens containing corticosteroids substantially increased the thrombogenic potential in these transplant recipients. Notably, all the assessed thrombotic risk markers (protein C, protein S, lupus anticoagulant, and antithrombin III) were within normal ranges. In addition, neither patient had any history of trauma or iatrogenic vascular injury. Therefore, no evidence of mechanical injury was identified in both cases.

Thrombus formation in renal transplant recipients is often precipitated by a primary etiological factor, with additional contributory factors exerting a synergistic effect. In the two reported cases, intraoperative findings revealed compression and stenosis of the common iliac vein, which likely served as the initial trigger for subsequent thrombosis. The angiographic evaluations demonstrated unobstructed renal veins with no evidence of stenosis or distortion. In these two cases, lower limb edemas prompted further investigation, leading to the detection of thrombosis within the transplanted renal veins. This observation raises a pertinent clinical question: Does isolated transplant renal vein thrombosis significantly contribute to graft failure? This underscores the importance of routine vascular patency assessments of transplanted renal arteries and veins as integral components of the diagnostic evaluation in cases of transplant dysfunction. Such screening is vital not only for prompt diagnosis and timely intervention to preserve graft function but also for safeguarding the patient's physical and psychological wellbeing.

Furthermore, the pathophysiological changes associated with such damage have seldom been researched by pathological or etiological studies, and there is a scarcity of related basic scientific research. In the two cases presented here, the course of renal vein thrombosis did not exceed 48 h. The thrombi were fresh, and no thrombolytic agents, such as urokinase, were administered prior to the AngioJet catheter thrombectomy. Instead, thrombus aspiration was performed directly for rapid clearance of the thrombus and gradual restoration of renal function. However, the post-procedure creatinine levels did not return to pre-thrombotic event levels, indicating that the thrombotic event led to acute kidney injury and a partial loss of renal function (12). The severity of this injury may be closely related to the time taken to restore the blood flow. Therefore, in kidney transplant patients, it is critical to remove the thrombus and restore blood flow as quickly as possible. With current technology, patients with uremia, with or without anticoagulant or thrombolytic therapy, are at higher risk for bleeding than those without renal impairment. Second, regarding the surgical approach, patients with uremia may not tolerate general anesthesia or the procedure itself, and thrombectomy of the transplanted renal vein can remove the thrombus from the main renal vein, but it is difficult to remove a thrombus from the branch veins. There is a greater risk to the overall physiological state of the patient, including a higher risk of anesthesia-related complications and a longer recovery time. AngioJet catheter thrombectomy is a minimally invasive surgical procedure that can achieve thrombus removal with less trauma to patients and reduce the risk of postoperative complications, leading to a shorter hospital stay (13). However, AngioJet is not applicable to all patients, and several studies have reported that AngioJet may cause bradyarrhythmia and hypotension or asymptomatic hemoglobinuria (13, 14). Therefore, the ATTRACT trial suggests that AngioJet thrombectomy may not benefit patients older than 65 years, and we need to explore more effective and safe methods in clinical practice (15, 16). For postoperative anticoagulation in both cases, we administered LMWH at 80 U/kg every 12 h (q12 h), based on the following rationale. First, the thrombi were in the acute phase, and the coagulation parameters indicated persistent hypercoagulability, suggesting a high risk of recurrence. Second, comprehensive bleeding risk assessments revealed low hemorrhagic potential in these patients. After carefully weighing these factors, we determined that full-dose LMWH remained the optimal anticoagulation approach for these cases.

## Conclusion

In cases of kidney transplant failure, the patency of the transplant renal arteries and veins should be routinely screened to prevent misdiagnosis. In prospective kidney transplant recipients, a pre-operative evaluation of the patency of the iliac vessels by a vascular surgeon is crucial. This can be assessed non-invasively through color Doppler ultrasound or MRI to reduce the risk of postoperative venous thrombosis affecting the transplant renal vein. Once a kidney transplant patient experiences a deep vein thrombosis event, it is crucial to promptly remove the thrombus and restore blood flow to minimize the extent of the acute kidney damage.

## Data Availability

The original contributions presented in the study are included in the article/Supplementary Material, further inquiries can be directed to the corresponding author.
